# Individual differences in Zhong-Yong tendency and processing capacity

**DOI:** 10.3389/fpsyg.2014.01316

**Published:** 2014-11-19

**Authors:** Ting-Yun Chang, Cheng-Ta Yang

**Affiliations:** Department of Psychology, National Cheng Kung UniversityTainan, Taiwan

**Keywords:** individual differences, linear ballistic accumulator model, systems factorial technology, workload capacity, Zhong-Yong

## Abstract

The present study investigated how an individual's Zhong-Yong tendency is related to his/her perceptual processing capacity. In two experiments, participants completed a Zhong-Yong Thinking Style Scale and performed a redundant-target detection task. Processing capacity was assessed with a non-parametric approach (systems factorial technology, SFT) and a parametric (linear ballistic accumulator model, LBA) approach. Results converged to suggest a positive correlation between Zhong-Yong tendency and processing capacity. High middle-way thinkers had larger processing capacity in multiple-signal processing compared with low middle-way thinkers, indicating that they processed information more efficiently and in an integrated fashion. Zhong-Yong tendency positively correlates with the processing capacity. These findings suggest that the individual differences in processing capacity can account for the reasons why high middle-way thinkers tend to adopt a global and flexible processing strategy to deal with the external world. Furthermore, the influence of culturally dictated thinking style on cognition can be revealed in a perception task.

## Introduction

People in different cultures differ psychologically, and they know different things, believe different things, and have different tastes. An increasing number of studies have investigated whether culture affects an individual's behavior and recent findings show that culture plays an important role in shaping human perception and cognition (Norenzayan and Nisbett, 2000; Masuda and Nisbett, 2001, 2006; Kitayama et al., 2003; Nisbett and Miyamoto, 2005; Miyamoto et al., 2006). Although it is still unclear whether this cultural influence is a result of collective unconsciousness, which is inherited through genes, or cumulative learning of the cultures, within-culture and cross-culture comparisons reveal the within- and between-cultural variation and reveal how human behavior is affected by social-cultural factors. The present study focuses on one of the most influential Chinese thinking styles, Zhong-Yong thinking style, to see how it affects the processes in perceptual decision making.

Middle-way thinking, also known as *Zhong-Yong* in Chinese, is a culturally dictated thinking style originating from Confucian philosophy. Being without inclination to either side is called *Zhong*; admitting of no change is called *Yong*. Zhong-Yong, the law of mind, was handed down from one to another in the Confucian school, until Tsze-Sze wrote a book chapter titled “*The Doctrine of the Mean*.” In *The Doctrine of the Mean*, the state of “equilibrium” and the state of “harmony” are emphasized and people are encouraged to achieve these mind states. In Chapter 1, Tsze-Sze states that “*While there are no stirrings of pleasure, anger, sorrow, or joy, the mind may be said to be in the state of equilibrium. When those feelings have been stirred, and they act in their due degree, there ensues what may be called the state of harmony. Equilibrium is the great root from which grow all the human acting in the world, and harmony is the universal path which they all should pursue*.” Also, written in the *Analects of Confucius*, the cognitive style of “middle-way” is described as the rule of thumb to deal with things and get along with other people. By a simplified definition, Zhong-Yong emphasizes that one should “… consider things carefully from different perspectives, avoid going to extremes, behave in situationally appropriate ways, and maintain interpersonal harmony…” (Ji et al., 2010). Middle-way thinking is regarded as a “good” individual attribute that the Chinese praise and pursue, and it has a major impact on Chinese daily life (see Yang, 2010 for a review).

Since C.-F. Yang and C.-Y. Zhao initiated a project to study different aspects of Zhong-Yong thinking in the early 1990s, an increasing number of studies have used the Zhong-Yong Thinking Style Scale (Chiu, 2000; Wu and Lin, 2005; Huang et al., 2012) to investigate the relationship between Zhong-Yong tendency and behavior. The results of these investigations converge to suggest that high middle-way thinkers tend to adopt a more global and flexible cognitive processing strategy when interacting with the external world. For example, in Huang et al. (in press) recent study, the researchers primed the participants with a neutral word or an emotional word prior to showing them a global-local stimulus on each trial. They found that the global precedence effect was larger for the high middle-way thinkers than the low middle-way thinkers only when emotion was primed. These results suggest that the global processing strategy, i.e., stepping back to see the whole picture, characterizes a high middle-way thinker's cognitive processing style. These results also imply that Zhong-Yong, served as an emotional regulator, affected an individual's cognitive processing strategy; this emotion regulation mechanism has not been reported in the previous models of emotion. In another study, Wang et al. (2013) examined how Zhong-Yong tendency is correlated with behavioral aspects of viewing banner ads. Participants were presented with banner ads of different levels of information complexity. The eye tracking data showed that high middle-way thinkers, compared to low middle-way thinkers, viewed banner ads of lower complexity with a larger and more distributed scan path, suggesting that they adopted a more global strategy to integrate information from all regions of the ads. In addition, high middle-way thinkers started to fixate on the banner ads of lower complexity at earlier time points. Wang et al. (2013) interpreted these findings as evidence that high middle-way thinkers were more efficient and flexible in switching from global processing (e.g., processing banner ads' gist) to local processing (e.g., processing banner ads' details).

Although the relationship between Zhong-Yong thinking style and cognitive processing style has been widely investigated, less is known about how an individual's perceptual processing capacity is related to his/her Zhong-Yong tendency. Perceptual processing capacity, also known as workload capacity, is defined as the change in processing efficiency of an information processing system that occurs as the workload (the number of to-be-processed signals) increases (Townsend and Nozawa, 1995; Wenger and Gibson, 2004; Eidels et al., 2011; Townsend and Eidels, 2011; Houpt and Townsend, 2012). Perceptual processing capacity is measured with a redundant-target detection task (Miller, 1978, 1982; Townsend and Nozawa, 1995), where participants monitor two sources of information and make a decision based on either one or both sources of information. If the processing speed of an individual channel is not affected by an increase in workload, the information processing system is defined as being unlimited in capacity; if the processing speed speeds up, the processing system is considered to have supercapacity; and lastly, if the processing speed slows down, the processing system is considered to have limited capacity. An individual's perceptual processing capacity is assumed to be independent of the way he/she processes information (Townsend and Nozawa, 1995); however, some multiple-signal processing strategies may be constrained by a system's processing capacity. For example, a coactive system usually has supercapacity, whereas the processing capacity of a standard serial system is limited (Townsend, 1972, 1974; Colonius and Townsend, 1997; Townsend and Nozawa, 1997; Wenger and Townsend, 2001; Wenger and Gibson, 2004; Eidels et al., 2011; Townsend and Eidels, 2011). In addition, a parallel system with supercapacity or limited capacity may imply that there are facilitatory or inhibitory between-channel interactions during the stage of information accumulation (Colonius and Townsend, 1997; Wenger and Gibson, 2004; Eidels et al., 2011). Thus, uncovering individual differences in perceptual processing capacity between high and low middle-way thinkers can help researchers understand the causes of differences in their cognitive processing styles.

The present study aimed to investigate the relationship between middle-way thinking style and perceptual processing capacity. In two experiments, participants completed the Zhong-Yong Thinking Style Scale (Wu and Lin, 2005) and performed a redundant-target detection task. We estimated the participants' perceptual processing capacity using a non-parametric approach (systems factorial technology, or SFT, see Townsend and Nozawa, 1995 for a review) in both experiments and a parametric approach (linear ballistic accumulator model, or LBA model, Brown and Heathcote, 2008; Eidels et al., 2010) in Experiment 2. These two approaches provide converging measures of workload capacity and have complementary advantages in the assessment (Eidels et al., 2010). We hypothesized that high middle-way thinkers tend to adopt a more global processing strategy to process information compared to low middle-way thinkers; thus, they process information in a more efficient way, especially when the workload increases, leading to supercapacity processing. On the other hand, low middle-way thinkers are more limited in perceptual processing capacity such that they are more prone to interference by information complexity.

## Experiment 1

In Experiment 1, a Go/No-go version of the redundant-target detection task was conducted to measure individuals' perceptual capacity for processing an object's color and shape. We used a non-parametric approach (SFT, see Townsend and Nozawa, 1995 for a review) to estimate perceptual processing capacity. The experimental design and data analysis followed the suggestions of SFT, which will be extensively described in the *Method* Section. The participants were split into two groups according to their Zhong-Yong scores, and the capacity coefficient of each group was plotted as a function of reaction time. We expected to observe qualitatively different capacity coefficient functions between high and low middle-way thinkers.

### Methods

#### Participants

Fifty-seven undergraduate students (29 males and 28 females) at National Cheng Kung University participated in this experiment. All participants had normal or corrected-to-normal vision, and their mean age was 20.63 years with a standard deviation of 2.72. Prior to the experiment, each participant signed a written informed consent, which has been proved by the review board of the National Cheng Kung University, Department of Psychology.

#### Apparatus

A personal computer with a 2.40 G-Hz Intel Pentium IV processor controlled the display and recorded the manual responses. The display resolution was 1024 × 768 pixels. Stimuli were presented on a 19-inch CRT monitor with a refresh rate of 85 Hz. The experiment was programmed with E-prime 1.1 (Schneider et al., 2002). The viewing distance was 60 cm. A chin-rest was used to prevent head movements.

#### Questionnaire

The participants' Zhong-Yong tendency was measured with a Zhong-Yong Thinking Style Scale, which was developed by Wu and Lin (2005). The Zhong-Yong Thinking Style Scale is composed of 13 items which are divided into three subscales that measure the three different aspects of Zhong-Yong, including diversification (i.e., considering things carefully from different aspects), integrity (i.e., integrating one's and others' perspectives), and harmony (i.e., acting in a manner for maintaining interpersonal harmony). Each item is scored on a 7-point Likert-type scale from “Strongly Disagree” (1) to “Strongly Agree” (7). An individual's Zhong-Yong score is defined as the mean score of the average scores of the three subscales. The Zhong-Yong score ranges from 1 to 7. Wu and Lin (2005) tested two samples in Studies 1 (*n* = 96) and 2 (*n* = 216) to measure the reliability and validity of the Zhong-Yong thinking style scale. They found that the coefficient of the internal consistency was 0.87 for both samples and the test-retest reliability was 0.81 (*n* = 46). The results of factor analysis showed that this scale is a single-factor scale and the factor loading for each item was greater than 0.40, suggesting that all the items are good measures of the construct of Zhong-Yong. In addition, Zhong-Yong score is positively correlated to self-consciousness, self-reflection, and inclusion of other in the self, showing high construct validity of the scale (Wu, 2006).

#### Design, stimuli, and procedure

**Figure 1:**
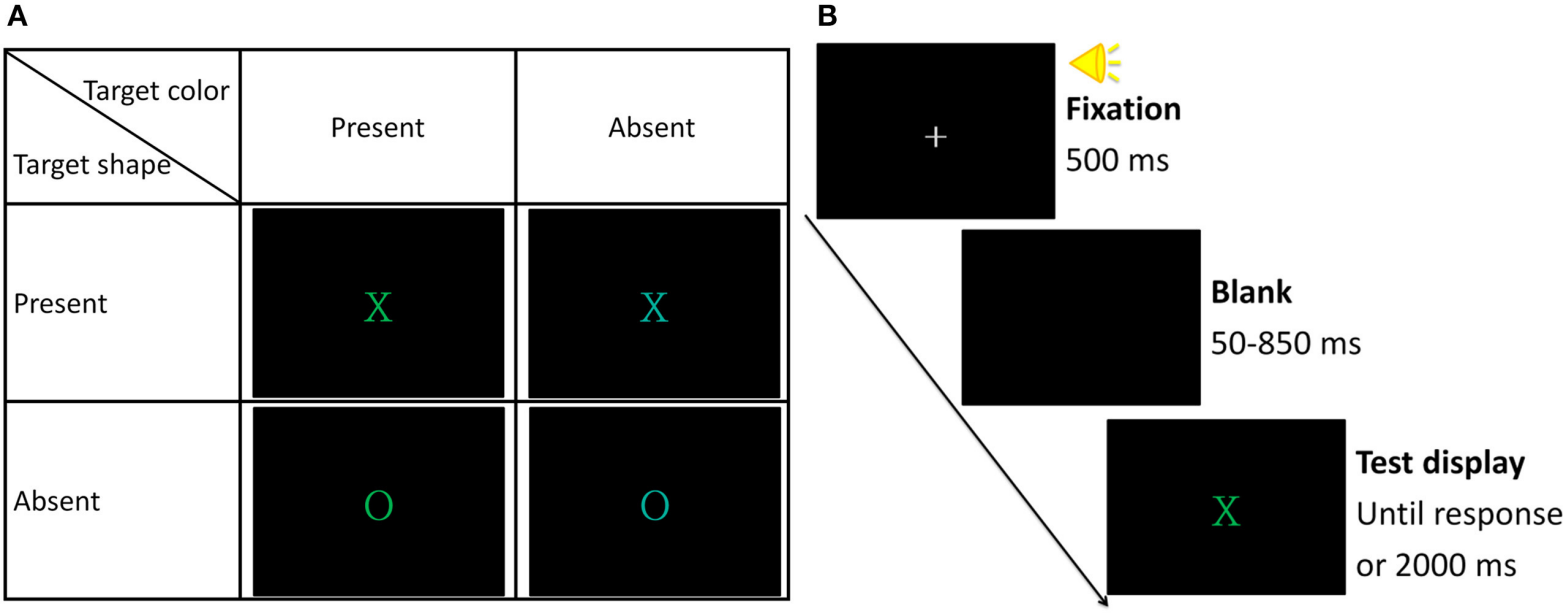
**(A)** Illustration of all possible test trials. **(B)** Illustration of the experimental procedure of the redundant-target detection task.

In the redundant-target detection task, each test display consisted of a colored letter (X or O) presented at the center of the screen. Its color was either green (*x* = 0.30, *y* = 0.60, *luminance* = 1.90 cd/m^2^) or cyan (*x* = 0.33, *y* = 0.33, *luminance* = 2.71 cd/m^2^). The size of the letter was 1° × 1°. The target color was defined as green and the target shape was defined as X; the distractor color was defined as cyan and the distractor shape was defined as O. The test display consisted of both target features (i.e., a green X, redundant-target condition), either target feature (i.e., a green O or a cyan X, single-target condition), or neither target feature (i.e., a cyan O, no-target condition) (see Figure 1A for all the possible test trials). Each condition was equally probable and was randomly intermixed within each block such that the participants would not anticipate the presence of the redundant-target trials (Mordkoff and Yantis, 1991, 1993). There were 40 practice trials and twelve blocks of 80 formal test trials in each experiment.

The experiment was conducted in a dimly lit room. A trial began with a 500 ms fixation cross, accompanied with a 750 Hz pure tone (see Figure 1B for an illustration of the experimental procedure). After a blank interval ranging from 50 to 850 ms, a test display was presented. Participants were instructed to press the “/” key if they detected either target feature (color green or shape X) and they were instructed to hold their responses if they detected neither target feature. The test display disappeared after a response was made (Go trial); otherwise, it remained on the screen until 2000 ms had passed (No-go trial). The inter-trial interval (ITI) was 500 ms. Both speed and accuracy were emphasized.

#### Data analysis

According to SFT, the capacity coefficient *C(t)* was computed to infer an individual's perceptual processing capacity. The capacity coefficient *C(t)* can be expressed as follows (Townsend and Nozawa, 1995; Townsend and Eidels, 2011; Houpt and Townsend, 2012; Houpt et al., 2014):

(1) $$C\left(\text{t}\right)=\frac{\log S_{1,2}(\text{t})}{\log\left[S_{1}\left(\text{t}\right)\cdot S_{2}\left(\text{t}\right)\right]},$$

for *t* > 0, where *S*_1_, *S*_2_, and *S*_1,2_ represent the survivor functions of the two single-target conditions and the redundant-target condition, respectively. The ranges of values of *C(t)* and their implications are as follows: if *C(t)* > 1, the system is supercapacity; if *C*(*t*) = 1, the system is unlimited-capacity; if *C(t)* < 1, it is limited-capacity; and if *C(t)* ≦ 0.5, the system is extremely limited in capacity.

### Results and discussion

We first analyzed the participants' Zhong-Yong tendency. The mean Zhong-Yong score for all of the participants was 5.80 with a standard deviation of 0.63. The participants were split into two groups according to their Zhong-Yong scores: the high middle-way thinkers (*N* = 10, *M* = 6.69, *SD* = 0.17) were the ones who scored at the top one-fifth on the Zhong-Yong scores and the low middle-way thinkers (*N* = 12, *M* = 4.93, *SD* = 0.32) were the ones who scored at the bottom one-fifth on the Zhong-Yong scores^1^. There was a significant difference in the Zhong-Yong scores between groups [*t*_(17.25)_ = 16.40, *p* < 0.0001]^2^.

Next, we examined the mean performance on the redundant-target detection task for each group of participants (see Table 1). Correct reaction times ranging from 150 to 1000 ms were extracted for further analysis. This range was chosen because simple reaction time is generally not faster than 150 ms and is not longer than 1000 ms. Under this criterion, a total of 1.4% data points were excluded from analysis. The mean accuracy was very high across conditions for both groups of participants except for the no-target conditions, suggesting a potential response bias in making a decision. We limited the remainder of our analyses to the reaction times. The mean reaction time in the redundant-target condition was faster than that in the single-target condition for the high middle-way thinkers [*t*_(9)_ = 12.30, *p* < 0.0001] and for the low middle-way thinkers [*t*_(11)_ = 3.47, *p* < 0.01], suggesting that the redundant-target effect was consistently found in both groups of participants. In addition, the redundancy gain was not significantly different between the groups [*t*_(13.16)_ = 0.42, *p* = 0.68].

**Table 1:** **Mean performance of the redundant-target detection task for each group of participants in Experiment 1**.

**Group**	**Accuracy**			**Reaction time (ms)**		
	***RT***	***ST***	***NT***	***RT***	***ST***	***RG***
High	0.99	0.99	0.86	397.79	441.64	43.85
Low	0.99	0.96	0.81	399.10	438.05	38.95

*“High” and “Low” denote the high and low middle-way thinkers, respectively. “RT,” “ST,” and “NT” represent the redundant-target, single-target, and no-target conditions, respectively. Redundancy gain (RG) is defined as the difference in mean reaction times between the redundant-target and single-target conditions. Note that mean reaction time of the no-target condition was not shown because in Experiment 1 any response in this condition is incorrect for the Go/No-go version of the redundant-target detection task*.

**Figure 2:**
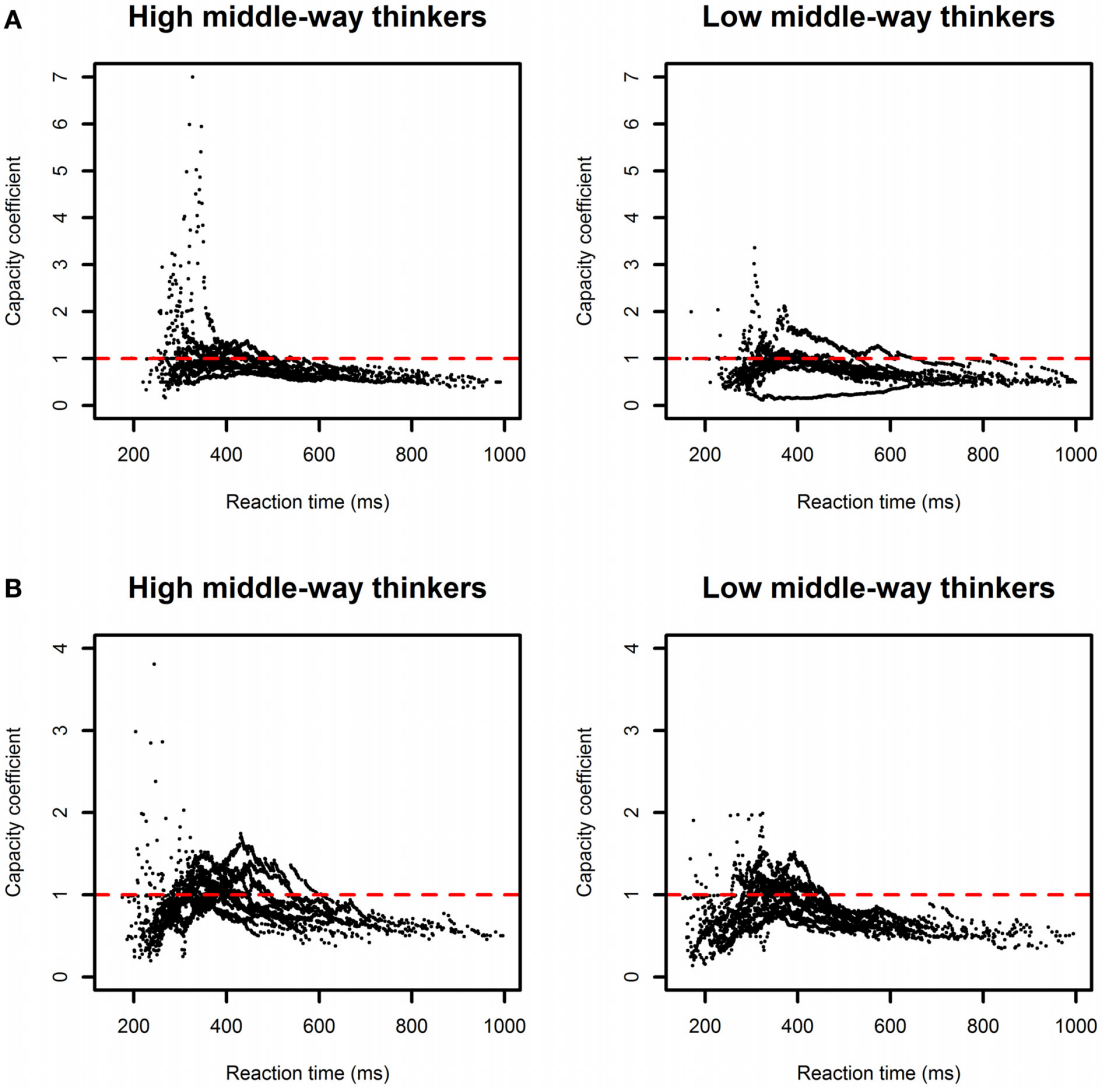
**(A)** Plots of the capacity coefficient *C(t)* for the high and low middle-way thinkers in Experiment 1. **(B)** Plots of the capacity coefficient *C(t)* for the high and low middle-way thinkers in Experiment 2.

We then computed *C(t)* for each participant and plotted the estimated *C(t)* by group. Figure 2A shows *C(t)* as a function of reaction time for each group. From visual inspection, the results showed that for most high middle-way thinkers, *C(t)* was larger than 1 for the faster reaction times, suggesting supercapacity processing. By contrast, for most low middle-way thinkers, *C(t)* was less than 1 for all times *t* and a few values of *C(t)* were hovering between ~0 and 0.5, suggesting limited-capacity to extremely limited-capacity processing. To verify these observations, we adopted a non-parametric bootstrapping method to simulate 1000 samples for each condition and to construct the 95% confidence interval for *C(t)* individually (Van Zandt, 2000). If the 95% confidence interval for *C(t)* exceeds 1 at some times *t*, we conclude that the participant adopts supercapacity processing to process multiple signals. Otherwise, we conclude that the participant adopts unlimited-capacity or limited-capacity processing. Table 2 presents the classification results of the inferences based on the simulated data for each group. Results showed that 4 out of 10 high middle-way thinkers adopted supercapacity processing; in contrast, only 1 (out of 12) low middle-way thinkers showed this pattern of results. When applying Fisher's exact test to test whether processing capacity and Zhong-Yong tendency are independent, the results, however, did not reach the significance level (*p* = 0.14). It is perhaps due to the small sample size that we did not obtain a significant result. Though, there is a trend showing that more high middle-way thinkers had a supercapacity system than low middle-way thinkers.

**Table 2:** **The classification results (frequency) of the inferences based on the simulated data for each group in Experiments 1 and 2**.

**Group**	**Experiment 1**		**Experiment 2**	
	**Supercapacity**	**Non-supercapacity**	**Supercapacity**	**Non-supercapacity**
High	4	6	6	7
Low	1	11	2	13

*“High” and “Low” denote the high and low middle-way thinkers, respectively*.

The results of Experiment 1 were consistent with our expectations. The high middle-way thinkers had systems with larger perceptual processing capacity than the low middle-way thinkers. The high middle-way thinkers generally exhibited supercapacity processing, suggesting that they adopted coactive processing to process multiple sources of information or that there were facilitatory between-channel cross-talks during the stage of information accumulation (Eidels et al., 2011). In contrast, the low middle-way thinkers exhibited limited-capacity or extremely limited-capacity processing when processing multiple signals, suggesting that they processed information in sequence or that there were inhibitory interactions between channels (Eidels et al., 2011). Therefore, the current findings provided empirical support for the notion that the high middle-way thinkers process redundant information more efficiently and in an integrative fashion, and the low middle-way thinkers were much more limited in capacity such that they serially processed multiple sources of information and were prone to interference as the workload increased.

## Experiment 2

In Experiment 1, we adopted a non-parametric approach (SFT) to estimate perceptual processing capacity, and the results of the visual inspection showed that the high middle-way thinkers had larger perceptual processing capacity than the low middle-way thinkers. However, there are a few limitations in Experiment 1. First, we only used correct reaction times for capacity estimation while ignoring the incorrect reaction times. Second, the lower accuracy in the no-target condition may reflect a potential response bias in target detection. Third, the extreme-group approach adopted in Experiment 1 only provides a discrete distinction between the high and low middle-way thinkers. It is unclear whether there is a linear relationship between Zhong-Yong tendency and perceptual processing capacity. Hence, a parametric approach, LBA model (Brown and Heathcote, 2008; Eidels et al., 2010), was adopted in Experiment 2 to estimate perceptual processing capacity in order to obtain a continuous measurement of the relationship between the Zhong-Yong tendency and perceptual processing capacity. This approach also provides researchers with a parametric testing tool to identify the perceptual processing capacity of a system. To implement the LBA model in this experiment, a yes/no version of the redundant-target detection task was used instead of a Go/No-go version of the redundant-target detection task because the analysis required reaction time data in both the target-present condition and the target-absent condition. We expected that the relationship between Zhong-Yong tendency and perceptual processing capacity observed in Experiment 1 would generalize to the choice reaction time experiment.

### Methods

#### Participants

Seventy-three undergraduate students (27 males and 46 females) at National Cheng Kung University who had not participated in Experiment 1 participated in this experiment. All of the participants had normal or corrected-to-normal vision, and their mean age was 19.27 years with a standard deviation of 1.34. Prior to the experiment, each participant signed a written informed consent, which has been proved by the review board of the National Cheng Kung University, Department of Psychology.

#### Design, stimuli, and procedure

The stimuli, design, and procedure used in the redundant-target detection task were the same as those in Experiment 1, except that the participants were instructed to make a yes/no response for target detection. When the participants detected either target feature, they had to press “/” key; otherwise, they had to press “z” key.

#### Data analysis

We used both a non-parametric approach (SFT, Townsend and Nozawa, 1995) as in Experiment 1 and a parametric approach (LBA model, Brown and Heathcote, 2008; Eidels et al., 2010) to estimate the participants' perceptual processing capacity. First, the estimated *C(t)* for the high and low middle-way thinkers were plotted separately and a non-parametric bootstrapping method was used to construct each participant's 95% confidence interval for *C(t)* to infer the perceptual processing capacity. Second, we computed the Pearson's product-moment correlation coefficient (*r*) between the LBA-based capacity and Zhong-Yong score to verify the relationship between the two measurements.

The following is a brief description of the LBA model (Brown and Heathcote, 2008; Eidels et al., 2010). The LBA model takes both correct and incorrect reaction times in the target-present and the target-absent conditions into consideration in the analysis. In a redundant-target detection task, four parallel accumulators are assumed to accumulate evidence independently and simultaneously about the presence of the target color (C), the absence of the target color (~C), the presence of the target shape (S), and the absence of the target shape (~S), respectively. Each accumulator starts to accumulate evidence from a random initial starting point, which is distributed as a uniform distribution in [0, *A*]. Evidence is accumulated linearly at a drift rate that is drawn from a normal distribution with a mean *v* and a standard deviation *s*. Accumulation is terminated and a decision is made when the amount of evidence reaches a threshold *b*. The reaction time is the decision time (i.e., the time for the accumulation reaching the threshold) plus the base time *t*_0_ (i.e., the time for the perceptual processing and motor execution).

In a redundant-target detection task, either of the yes/no responses can be made on each trial: “*YES*” for the presence of either target feature, and “*NO*” for the absence of both target features. Specifically, a “*YES*” response occurs when accumulator C reaches the threshold but accumulator S has not reached the threshold or when accumulator S reaches the threshold but accumulator C has not reached the threshold. The overall likelihood of a “*YES*” response occurring at time *t* is expressed as

(2) $$\begin{array}{l} \text{L}\left(\text{YES},\text{t}\right)=\left[1-{\text{F}}_{\sim \text{C}}\left(t\right)\cdot {\text{F}}_{\sim \text{S}}\left(t\right)\right]\cdot [f_{C}\left(t\right)\cdot {\text{S}}_{\text{S}}\left(t\right)\\ \;+f_{S}\left(t\right)\cdot {\text{S}}_{\text{C}}\left(t\right)], \end{array}$$

where *F*, *f*, and *S* denote the cumulative distribution function, density function and survivor function for each accumulator, respectively. Similarly, a “*NO*” response occurs when accumulators ~C and ~S reach the threshold before accumulators C and S have not reached the threshold. The overall likelihood of a “*NO*” response occurring at time *t* is expressed as

(3) $$\begin{array}{l} \text{L}\left(\text{NO},\text{t}\right)={\text{S}}_{\text{C}}\left(t\right)\cdot {\text{S}}_{\text{S}}\left(t\right)\cdot [f_{\sim C}\left(t\right)\cdot {\text{F}}_{\sim \text{S}}\left(t\right)\\ \;+f_{\sim S}\left(t\right)\cdot {\text{F}}_{\sim \text{C}}\left(t\right)]. \end{array}$$

Likelihood functions, L(*YES*,*t*) and L(*NO*,*t*), were used to obtain the maximum likelihood estimates of the parameters for each accumulator given the correct and incorrect reaction times. The initial starting point *A* was fixed across conditions, and the standard deviation *s* was set as 0.25 in reference to Donkin et al. (2009). We assumed two decision threshold parameters for the target-present condition (*b_T_*) and target-absent condition (*b_NT_*) because the participants may set different criteria for making “*YES*” and “*NO*” responses due to the unequal presentation probability across the two conditions. However, *b_T_* was assumed to not vary across the redundant-target condition and the two single-target conditions because changes in the boundary parameter were unlikely to occur when all target-present conditions were randomly intermixed within a block (Ratcliff, 1978). Base times for the redundant-target accumulator (*t*_0*RT*_), the single-target accumulator (*t*_0*ST*_), and the no-target accumulator (*t*_0*NT*_) were estimated separately because sensory encoding time may vary as a function of the number of signals to be processed.

Drift rate estimation is the most important part of the estimation of the LBA-based capacity measure. When the target was present, we assumed three drift rate parameters for the redundant-target accumulator (*v_RT_*), the single-target accumulator (*v_ST_*), and the no-target accumulator (*v_NT_*). When the target was absent, we assumed two drift rate parameters for the no-target accumulator (*v*_~*NT*_) and the target accumulator (*v*_~*T*_). Note that there are 16 possible drift rate parameters (see Table 3), but we only estimated five of them because we assumed that the drift rates for accumulator C and accumulator S were the same and the drift rates for accumulator ~C and accumulator ~S were also the same. These two assumptions need not to be true; however, similar pattern of results was observed when we allowed the variation between all the 16 drift rate parameters. Therefore, a total of 11 free parameters (*A*, *b_T_*, *b_NT_*, *t*_0*RT*_, *t*_0*ST*_, *t*_0*NT*_, *v_RT_*, *v_ST_*, *v_NT_*, *v*_~*T*_, *v*_~*NT*_) were estimated for each participant.

**Table 3:** **The simplified set of five drift rate parameters (right-hand side) used in the LBA model and their corresponding drift rates of all accumulators (left-hand side) in the redundant-target task**.

		**Target color**	
		**Present (C)**	**Absent (~C)**
	Present (S)	*v*_*C*|*CS*_ = *v_RT_*	*v*_*C*|~*CS*_ = *v*_~*T*_
		*v*_*S*|*CS*_ = *v_RT_*	*v*_*S*|~*CS*_ = *v_ST_*
		*v*_~*C*|*CS*_ = *v_NT_*	*v*_~*C*|~*CS*_ = *v*_~*NT*_
		*v*_~*S*|*CS*_ = *v_NT_*	*v*_~*S*|~*CS*_ = *v_NT_*
Target shape			
	Absent (~S)	*v*_*C*|*C*~*S*_ = *v_ST_*	*v*_*C*|~*C*~*S*_ = *v*_~*T*_
		*v*_*S*|*C*~*S*_ = *v*_~*T*_	*v*_*S*|~*C*~*S*_ = *v*_~*T*_
		*v*_~*C*|*C*~*S*_ = *v_NT_*	*v*_~*C*|~*C*~*S*_ = *v*_~*NT*_
		*v*_~*S*|*C*~*S*_ = *v*_~*NT*_	*v*_~*S*|~*C*~*S*_ = *v*_~*NT*_

*Subscripts for the simplified set of five drift rates are described in the Data Analysis section of Experiment 2. Subscripts for the full set of 16 drift rate parameters denote the drift rate for a specific accumulator given any of the four test trials. For instance, v_C|CS_ represents the drift rate for accumulator C when both the target color and shape are present and is mapped to the drift rate for the redundant-target accumulator v_RT_*.

The LBA-based capacity is defined as the relative magnitudes between drift rates in the redundant-target condition and the single-target condition, which can be expressed as

(4) $$v_{\text{diff}}=v_{\text{RT}}-v_{\text{ST}}.$$

If *v_diff_* > 0, the system is supercapacity processing; if *v_diff_* = 0, the system is unlimited-capacity processing; if *v_diff_* < 0, the system is limited-capacity processing.

### Results and discussion

Data from two participants were excluded because they were unable to follow the experimental instructions. The mean Zhong-Yong score for all of the participants was 5.72 with a standard deviation of 0.70. We used an extreme-group approach, as we did in Experiment 1. The participants who scored at the top one-fifth on the Zhong-Yong score were regarded as high middle-way thinkers (*N* = 13, *M* = 6.56, *SD* = 0.16), and the participants who scored at the bottom one-fifth on the Zhong-Yong score were considered as low middle-way thinkers (*N* = 15, *M* = 4.67, *SD* = 0.58). There was a significant difference in the Zhong-Yong scores between groups [*t*_(16.31)_ = 12.13, *p* < 0.0001].

Next, we examined the mean performance of the redundant-target detection task for each group of participants (see Table 4). Using the same criterion as Experiment 1, a total of 6.1% reaction time data of the redundant-target detection task was excluded from further analysis. Similar to Experiment 1, accuracy was lower in the no-target conation than the other conditions, suggesting a potential response bias in target detection. Although the mean performance in this experiment was worse than that in Experiment 1 [accuracy: *t*_(105.40)_ = 2.06, *p* < 0.05; reaction time: *t*_(114.70)_ = 10.89, *p* < 0.0001], we still observed the redundant-target effect for both the high middle-way thinkers [*t*_(12)_ = 10.76, *p* < 0.0001] and the low middle-way thinkers [*t*_(14)_ = 10.04, *p* < 0.0001. In addition, the redundancy gain was not significantly different between the groups [*t*_(25.33)_ = 1.14, *p* = 0.27].

**Table 4:** **Mean performance of the redundant-target detection task for each group of participants in Experiment 2**.

**Group**	**Accuracy**			**Reaction time (ms)**			
	***RT***	***ST***	***NT***	***RT***	***ST***	***NT***	***RG***
High	0.99	0.96	0.88	348.81	396.14	457.98	47.33
Low	0.99	0.96	0.83	362.99	403.53	477.95	40.54

*“High” and “Low” denote the high and low middle-way thinkers. “RT,” “ST,” and “NT” represent the redundant-target, single-target, and no-target conditions, respectively. Redundancy gain (RG) is defined as the difference in the mean reaction times between the redundant-target and single-target conditions*.

As in Experiment 1, we computed *C(t)* and constructed the 95% confidence interval for *C(t)* for each participant to infer the perceptual processing capacity. Figure 2B plots the results of *C(t)* for each group of participants. The results of the non-parametric measures of capacity replicated what we found in Experiment 1; that is, *C(t)* was generally larger for the high middle-way thinkers than for the low middle-way thinkers. Based on the simulated data (see Table 2), we inferred that 6 out of 13 high middle-way thinkers had a system of supercapacity processing, while only 2 out of 15 low middle-way thinkers showed this pattern of results. Note that a few low middle-way thinkers had *C(t)* that was greater than 1 at early time points (see Figure 2B); however, compared to high middle-way thinkers, the values of *C(t)* were relatively small, suggesting that low middle-way thinkers were less efficient in processing multiple sources of information. We then conducted a Fisher's exact test to test whether processing capacity and Zhong-Yong tendency are independent. The result still did not reach the significance level (*p* = 0.10) although there is a trend showing that more high middle-way thinkers were classified in the supercapacity category than low middle-way thinkers and less high-middle-way thinkers were classified in the non-supercapacity category than low middle-way thinkers. Nevertheless, when we combined the data of Experiments 1 and 2 to increase the sample size, the result of the Fisher's exact test was significant (*p* < 0.05), verifying that Zhong-Yong tendency and processing capacity are dependent on each other.

**Figure 3:**
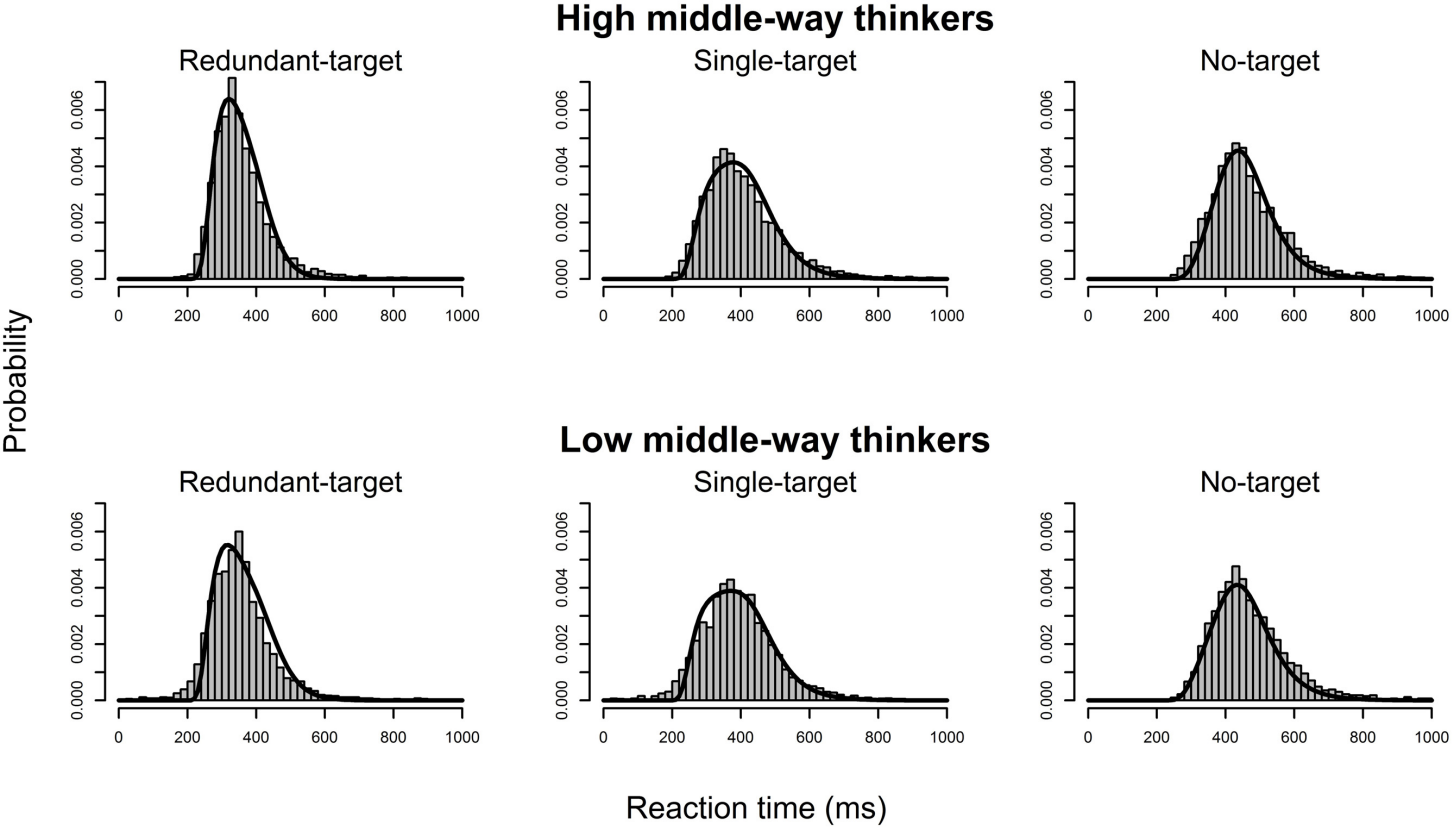
**Plots of the predicted density functions on top of the empirical reaction time histograms of the redundant-target, single-target, and no-target conditions for each group**.

Next, we adopted the LBA model to analyze the reaction time data to estimate a set of parameters that maximized the likelihood function described in the *Method* Section for each participant. Table 5 presents the average of 11 estimated parameters for each group. None of the parameters differed between high and low middle-way thinkers (*p*s > 0.12). We then used the average of the estimated parameters to generate model predictions from the LBA model and plotted the empirical histograms for correct responses along with corresponding model predictions (see Figure 3). The results showed that the LBA model successfully captured the underlying distributions of the reaction time data, suggesting that the LBA model fit the participants' reaction time data well.

**Table 5:** **The average values of 11 estimated parameters and the LBA-based capacity (*v_diff_*) for the high and low middle-way thinkers**.

**Group**	**Estimated parameters**											
	***A***	***b_T_***	***b_NT_***	***t*_0*RT*_**	***t*_0*ST*_**	***t*_0*NT*_**	***v_RT_***	***v_ST_***	***v_NT_***	***v*_~*T*_**	***v*_~*NT*_**	***v_diff_***
High	288.06	507.53	581.60	112.59	101.32	79.45	1.29	1.22	0.66	0.37	1.34	0.07
Low	313.75	493.60	576.45	120.25	114.32	78.51	1.17	1.21	0.64	0.35	1.31	−0.04

*“High” and “Low” denote the high and low middle-way thinkers*.

**Figure 4:**
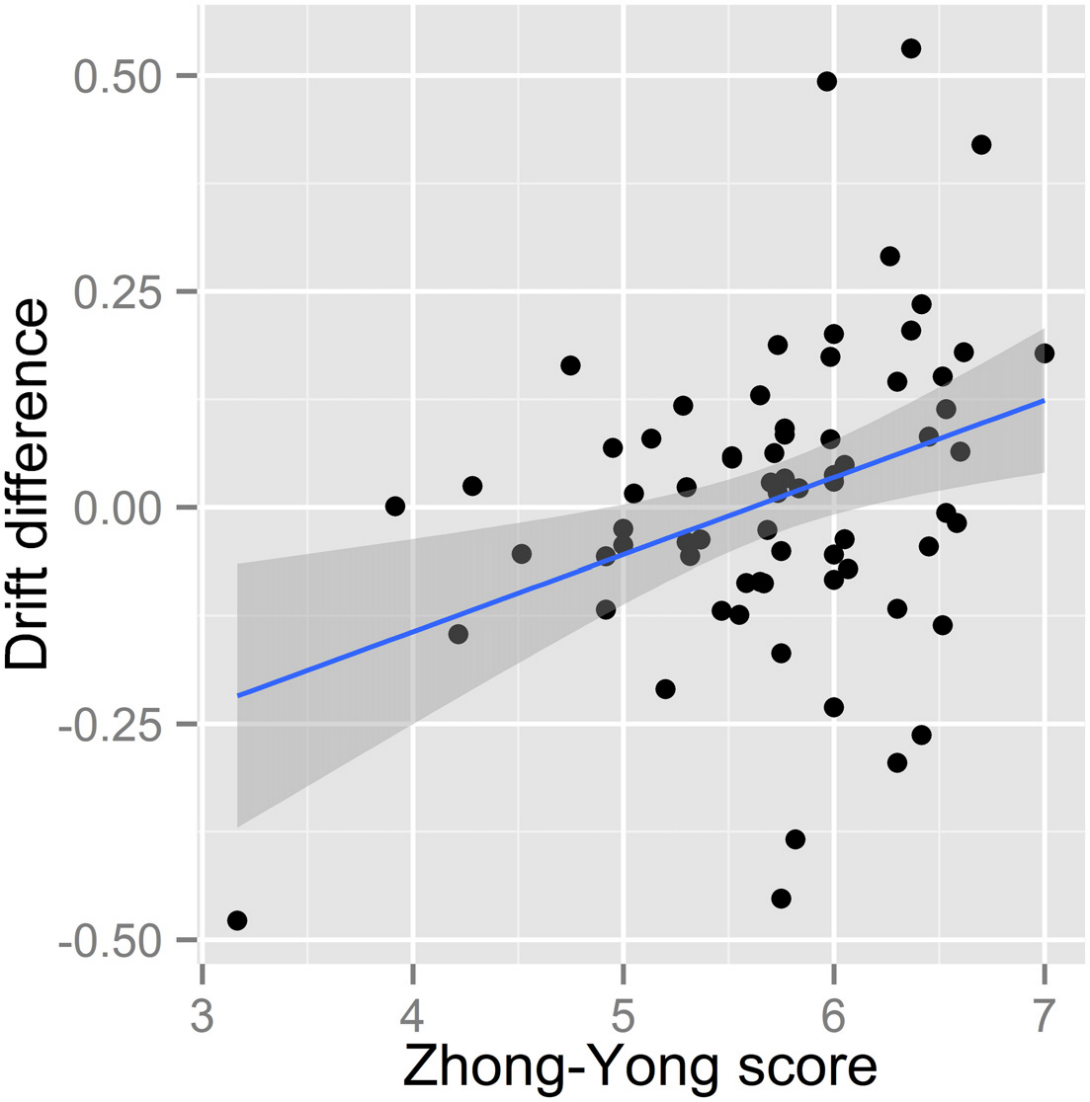
**Scatter plot of the drift difference and Zhong-Yong score with a trend line (solid blue line) and the 95% confidence interval for the trend (band-shaped gray area)**.

We then computed the LBA-based capacity (*v_diff_*) for each group (see Table 5). The results showed that the drift difference for the high middle-way thinkers (*M* = 0.07, *SD* = 0.17) was larger than that of the low middle-way thinkers (*M* = −0.04, *SD* = 0.16) [*t*_(24.40)_ = 1.87, *p* < 0.05]. Lastly, we computed the Pearson's product-moment correlation (*r*) between the LBA-based capacity and the Zhong-Yong score, and we found a significant positive correlation between the two measurements [*r* = 0.35, *p* < 0.01, 95% CI = (0.13, 0.54)] (Figure 4), suggesting that the perceptual processing capacity monotonically increases as Zhong-Yong tendency increases.

## General discussion

In the present study, two experiments were conducted to investigate how an individual's Zhong-Yong tendency is related to his/her perceptual processing capacity. The Zhong-Yong Thinking Style Scale (Wu and Lin, 2005) was used to assess the participant's Zhong-Yong tendency. The redundant-target detection task was adopted to infer the participants' perceptual processing capacity in a non-parametric manner (SFT in Experiments 1 and 2) as well as in a parametric manner (LBA model in Experiment 2). The results from the non-parametric and parametric analyses converged to suggest that participants with a strong Zhong-Yong tendency had larger perceptual capacity in processing redundant information for decision making. High middle-way thinkers had an unlimited-capacity to supercapacity processing system, suggesting that the processing time of an individual channel was unaffected or even sped up when workload increased. In contrast, low middle-way thinkers had a limited-capacity processing system, suggesting that the individual-channel processing time slowed down as a result of the increasing workload.

### Zhong-yong tendency and perceptual processing capacity

The current results were consistent with our expectation that high middle-way thinkers have larger perceptual processing capacity and process multiple signals more efficiently as workload increases. Two possible accounts may explain the reasons why the high middle-way thinkers had larger perceptual processing capacity than the low middle-way thinkers. First, it is worthwhile to note that although the processing architecture (i.e., the way that redundant information is processed) and the processing capacity (i.e., the variation in the efficiency of a system as a function of workload) are independent measures of information processing (Townsend and Nozawa, 1995), processing capacity may constrain the processing order of multiple signals. For example, a coactive system is commonly assumed to have supercapacity, while a standard serial model is assumed to be limited in capacity, although the standard serial model and the unlimited-capacity parallel model can mimic each other theoretically(Townsend, 1972, 1974; Colonius and Townsend, 1997; Townsend and Nozawa, 1997; Wenger and Townsend, 2001; Wenger and Gibson, 2004; Eidels et al., 2011; Townsend and Eidels, 2011). Our results showed that the high middle-way thinkers had supercapacity processing, implying that they tended to process redundant information in a coactive fashion. That is, multiple signals are processed in parallel and simultaneously, and separate activations from multiple channels are accumulated and summed into a single accumulator. A decision is made when the accumulated evidence reaches the decision criterion. By contrast, the low middle-way thinkers exhibited limited-capacity processing, implying that they had less capacity for multiple-signal processing such that they may process redundant information in a serial fashion. Namely, one of the target features is processed first, and if the information is sufficient for decision making, the other processing is terminated as predicted by a serial self-terminating model.

However, individual differences in perceptual processing capacity do not necessarily mean that high and low middle-way thinkers adopt different processing strategies. Assuming that multiple signals are processed in a parallel fashion for all participants, differences in processing capacity may suggest differences in the way multiple processes interact with each other during information accumulation. According to Eidels et al. (2011), different types of between-channel interactions explain the variation in the processing efficiency of an individual channel as workload increases. They simulated a parallel model with different levels of between-channel interactions and found that a parallel model with supercapacity processing suggests that there are facilitatory (positive) interactions between channels during information accumulation, while a parallel model with limited-capacity processing suggests that there are inhibitory (negative) between-channel cross-talks. Accordingly, high middle-way thinkers can integrate multiple signals more efficiently with positive between-channel interactions; by contrast, low middle-way thinkers are more prone to interference by information complexity due to negative between-channel interactions that result in mutual inhibitions between each process.

Future studies are required to further examine the possibility that high and low middle-way thinkers may adopt different multiple-signal processing strategies for decision making. An ongoing study has been designed following Townsend and Nozawa (1995) suggestions to use a standard double factorial paradigm in which nine test stimuli with simultaneous manipulation of the target feature and the target intensity are used to directly test the processing architecture adopted by high and low middle-way thinkers. In addition, this study may also enable us to uncover differences in between-channel interactions during information accumulation.

### Zhong-yong tendency and cognitive processing style

Many researchers are interested in understanding how culture shapes behavior. In regard to middle-way thinking, or Zhong-Yong, Chinese culture has long regarded middle-way thinking as one of the most important meta-cognitive factors that regulate one's emotions and attitudes (Ji et al., 2010; Yang, 2010). People who have a strong Zhong-Yong tendency can be characterized by their global and flexible cognitive processing styles (Wang et al., 2013; Huang et al., in press). In addition, a recent study showed that Zhong-Yong can moderate the relationship between perceived creativity and innovation behavior in Chinese companies (Yao et al., 2010).

The present study, which tested individual differences in perceptual processing capacity, can offer further insights into aspects of how Chinese culture influences individuals' behavior. First, individual differences can be observed in a relatively fundamental perceptual task (i.e., the color-shape detection task used in the present study). These findings are in line with previous research on cross-cultural comparisons between East Asian and West Caucasian (Norenzayan and Nisbett, 2000; Masuda and Nisbett, 2001, 2006; Kitayama et al., 2003; Nisbett and Miyamoto, 2005; Miyamoto et al., 2006). One distinction that has been revealed in cross-cultural research is the contrast between individualist cultures (Western culture) and collectivist cultures (Eastern culture) (see Triandis, 1995). Individualists emphasize individual achievements and goal; collectivists emphasize group membership and value group cohesion and success above personal achievement. Nisbett and colleagues conducted a large body of research, which suggests that members of individualist and collectivist cultures tend to have measurably different cognitive processing styles. That is, East Asians (collectivist) are field-dependent, and they process information more holistically, seeing the relation between things; by contrast, West Caucasians (individualist) are field-independent and they process information analytically, focusing on individual objects. The cultural variation in cognition and perception allows us to challenge the idea that the rules used in thought are fixed by a hard-wired mental logic and provides empirical supports for the top-down influence on perception.

Second, the current findings oppose the argument proposed by a few Zhong-Yong studies that the mechanism of Zhong-Yong thinking, the wisdom of “middle way,” is akin to the mechanism of Western wisdom, and its influence can be revealed only when conflicts, dilemmas, or affections are raised (Grossmann et al., 2010, 2013). This argument was empirically supported by Huang et al. (in press), in which differences were found in the global precedence effect between high and low middle-way thinkers only when participants' emotions were primed. Nonetheless, in the present study, we found individual differences in a perceptual task without manipulating emotions. One possibility to explain the inconsistent findings is the difference between the scales used in the current study and Huang et al.'s study. In the current study, we used the scale developed by Wu and Lin (2005) which measures three aspects of Zhong-Yong; by contrast, Huang et al. used the Zhong-Yong Belief-Value Scale developed by Huang et al. (2012) which emphasizes the harmony dimension of Zhong-Yong. Therefore, we suggest that the influence of Zhong-Yong can be context independent in terms of the way Zhong-Yong tendency is assessed. The culturally induced wisdom or thinking style is a stable meta-cognitive factor that regulates one's behavior and is not specific to any context. Perceptual processing capacity may play an important role in mediating the influence of Zhong-Yong thinking on cognitive processing style. Future investigations are required to verify the mediating role of perceptual capacity in dealing with complex cognitive tasks.

### Advantages and limitations of the present study

The present study adopted both parametric (LBA model) and non-parametric (SFT) mathematical modeling approaches to study individual differences in perceptual processing capacity, and both levels of analyses showed similar patterns of results. Compared to previous research that tested mean reaction time by aggregating the data of each group (Wang et al., 2013; Huang et al., in press), this study considered the reaction time distribution and inferred the information processing characteristics individually. In addition, SFT and the LBA model have compensatory advantages in analyzing reaction time distributions (Eidels et al., 2010). SFT only considers correct reaction time data but allows researchers to examine the processing architecture (serial vs. parallel vs. coactive), the decisional stopping rule (self-terminating vs. exhaustive), and the processing capacity (limited-capacity vs. unlimited-capacity vs. supercapacity) (Townsend and Nozawa, 1995). By contrast, the LBA model assumes that two processes occur in a parallel fashion, but it incorporates reaction time and accuracy data into the analysis (Brown and Heathcote, 2008; Eidels et al., 2010). In addition, the LBA model provides a statistical basis for making inferences about the perceptual processing capacity of an information processing system (Eidels et al., 2010).

However, testing the processing capacity does not directly test the processing order of multiple-signal processing, given that the perceptual capacity and the processing architecture are two independent measures of information processing (Townsend and Nozawa, 1995). To further understand how middle-way thinking influences information processing strategies, a standard double factorial paradigm (Townsend and Nozawa, 1995) is required, as stated in the previous section. With a closer examination of the variation of the processing characteristics of information processing, we can further our understanding of cultural differences in cognitive processing.

## Conclusion

The present study is the first study to elucidate the relationship between Zhong-Yong tendency and perceptual processing capacity. We found that individual differences in perceptual processing capacity are predicted well by an individual's Zhong-Yong tendency. Specifically, participants with stronger Zhong-Yong tendencies had larger perceptual processing capacities. These individual differences provide insight into the reasons why high middle-way thinkers are more flexible and efficient in processing multiple sources of information in an integrative fashion. These results emphasize that culture can shape an individual's cognitive processing style, and that the cultural shaping of cognitive style can be revealed in a fundamental perceptual task.

### Conflict of interest statement

The authors declare that the research was conducted in the absence of any commercial or financial relationships that could be construed as a potential conflict of interest.

## Footnotes

^1^The reason why we adopted the extreme-group approach is to emphasize the differences between high and low Zhong-Yong groups since the SFT results were somewhat noisy. However, even when we used median-split to analyze the data, we still obtained a similar pattern of results.

^2^We thank the anonymous reviewer for raising this question: both high and low Zhong-Yong groups show Zhong-Yong tendency even though there are significant differences in their Zhong-Yong scores. Unfortunately, there is no norm for the Zhong-Yong Thinking Style Scale. Therefore, we used the data reported in Wu and Lin (2005) to estimate the mean and standard deviation of their participants' Zhong-Yong scores (*n* = 216 in Study 2). The mean is 5.44 and the standard deviation is 0.32. Compared to our current findings [high middle-way thinkers: *M* = 6.69, *SD* = 0.17; low middle-way thinkers: *M* = 4.93, *SD* = 0.32], our high/low Zhong-Yong group had the score significantly higher/lower than the average score reported in the original study. Therefore, we can claim that the high and low Zhong-Yong groups in the current study had different Zhong-Yong tendency than the average of the Taiwanese population, although it is still possible that all the Taiwanese participants have a stronger Zhong-Yong tendency than other people from different culture backgrounds. Future studies are required to explore the cross-cultural variation.
